# New Vehicle Detection Method with Aspect Ratio Estimation for Hypothesized Windows

**DOI:** 10.3390/s151229838

**Published:** 2015-12-09

**Authors:** Jisu Kim, Jeonghyun Baek, Yongseo Park, Euntai Kim

**Affiliations:** 1 The School of Electrical and Electronic Engineering, Yonsei University, Seoul 120-749, Korea; jisukim2000@yonsei.ac.kr (J.K.); jhyun25@yonsei.ac.kr (J.B.); 2 Department of Electrical Engineering, Gachon University, Seongnam 461-701, Korea; yspark@gachon.ac.kr

**Keywords:** vehicle detection, ACF, ROI estimation

## Abstract

All kinds of vehicles have different ratios of width to height, which are called the aspect ratios. Most previous works, however, use a fixed aspect ratio for vehicle detection (VD). The use of a fixed vehicle aspect ratio for VD degrades the performance. Thus, the estimation of a vehicle aspect ratio is an important part of robust VD. Taking this idea into account, a new on-road vehicle detection system is proposed in this paper. The proposed method estimates the aspect ratio of the hypothesized windows to improve the VD performance. Our proposed method uses an Aggregate Channel Feature (ACF) and a support vector machine (SVM) to verify the hypothesized windows with the estimated aspect ratio. The contribution of this paper is threefold. First, the estimation of vehicle aspect ratio is inserted between the HG (hypothesis generation) and the HV (hypothesis verification). Second, a simple HG method named a signed horizontal edge map is proposed to speed up VD. Third, a new measure is proposed to represent the overlapping ratio between the ground truth and the detection results. This new measure is used to show that the proposed method is better than previous works in terms of robust VD. Finally, the Pittsburgh dataset is used to verify the performance of the proposed method.

## 1. Introduction

Vehicle detection (VD) is one of the major research issues within intelligent transportation system (ITS) organizations, and considerable research has been conducted. Most of the research works consist of two steps: HG (hypothesis generation) and HV (hypothesis verification). 

Concerning the HG, symmetry [1,2], color [3,4], shadow [5,6] and edges [7,8] were used to select the vehicle candidates. Further, search space reduction methods were developed to save the computational resources in HG. For example, in [9], the linear model between the vehicle position and vehicle size is updated using a recursive least square algorithm. This linear model helps to generate the Region of interests (ROIs) such that they are likely to include vehicle regions. Therefore, this approach can reduce false positives as compared with the previous exhaustive search or sliding window approaches. Interestingly, in [10], image inpainting is used to verify the detection results. Image inpainting is actually a method for restoring damaged images. This approach also reduces false positives. 

Concerning the HV, lots of research has focused on the application of machine vision technologies to VD, as in [11,12]. The HV works based on machine vision mainly consist of features and classifiers. In case of the features, the Histogram of oriented gradient (HOG) [13], Haar-like wavelet [14], Gabor feature [15] and Aggregate channel features (ACF) [16] are generally used. The Haar-like wavelet takes less computational time than the HOG or Gabor feature. However, the detection performance using the Haar-like wavelet is lower than that of the HOG or Gabor feature. In [17], the HOG and Haar-like wavelet are combined in cascade form to reduce the computational time and to improve the detection performance. The Haar-like wavelet accelerated the hypothesis generation (HG), while HOG verified the generated hypotheses. In addition to these features, the Gabor feature is also an effective feature for VD. The Gabor filter is a kind of band-pass filter that extracts specific information called the Gabor feature from the frequency domain. In [18], a gamma distribution is used to represent the Gabor feature for better detection performance when compared with the Gaussian distribution. The Gabor feature, however, takes a long computational time due to how the computation of a convolution is required. Some works have been reported [19,20] to reduce the computational time of the Gabor filter. In case of the classifiers, the support vector machine (SVM), Adaboost [21] and Neural Network (NN) are used to train the various features. Recently, Latent SVM has been researched for a deformable part-based model (DPM) [22]. This model can capture significant deformation in the object appearance.

On the other hand, all kinds of vehicles have different ratios of width to height, which are called the aspect ratios. Most previous works, however, use a fixed aspect ratio for vehicle detection (VD). The use of a fixed vehicle aspect ratio [23,24,25,26] for VD degrades the performance. Thus, a new step named HI (hypothesis improvement) is developed to enhance the VD performance in this paper. In the HI, the aspect ratio of the hypothesized windows is estimated and the result is applied to the classifier in the HV. Thus, the HI is positioned between the HG and the HV. A part of this paper was presented in [27]. The contribution of this paper is threefold: (1) the HI based on the estimation of vehicle aspect ratio is inserted between the HG (hypothesis generation) and the HV (hypothesis verification); (2) a simple HG method named a signed horizontal edge map is proposed to speed up VD; (3) a new measure is proposed to quantify how well the detection result matches the ground truth. This measure can be used to show that the proposed method is better than previous methods in terms of robust VD.

The remainder of this paper is organized as follows: In Section 2, the proposed vehicle detection system is briefly outlined. In Section 3, the vehicle aspect ratio is estimated and is used to generate an efficient ROI. In Section 4, some experiments are conducted to verify the validity of the proposed method. Some conclusions are drawn in Section 5.

## 2. Motivation and System Overview 

**Figure 1:**
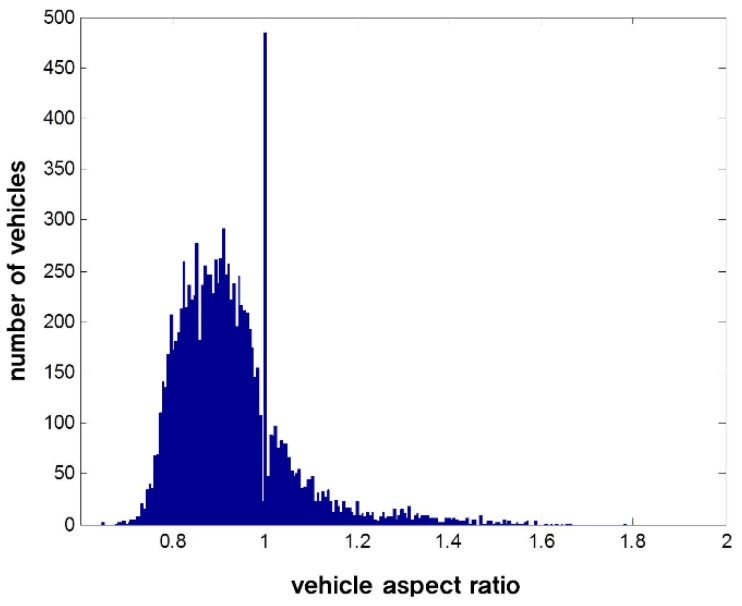
The distribution of vehicle aspect ratios in the Pittsburgh dataset.

Figure 1 shows the distribution of vehicle aspect ratios in the Pittsburgh dataset.

**Figure 2:**
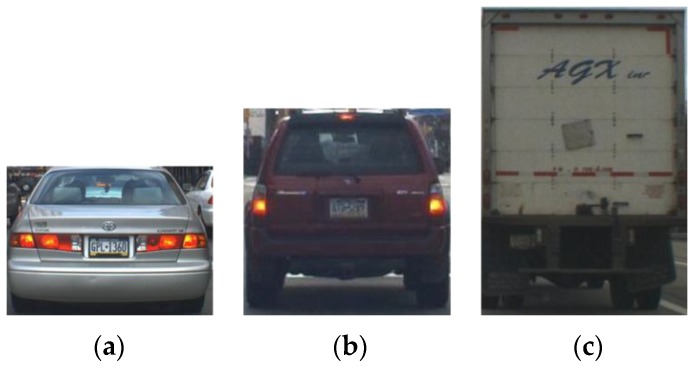
Vehicle images for various vehicle aspect ratios: (**a**) 0.7; (**b**) 1; and (**c**) 1.5.

A total of 10,907 vehicles are used in the Pittsburgh dataset, as shown in Figure 1. As can be seen in the figure, the vehicle aspect ratio is approximately 1, but it varies from 0.5 to 2, depending on the types of vehicles and the camera viewpoint. Examples of vehicle images are given in Figure 2. In general, sedans have low vehicle aspect ratios, while trucks or buses have high vehicle aspect ratios, as shown in Figure 2. Thus, the use of a fixed aspect ratio in VD can degrade the performance.

**Figure 3:**
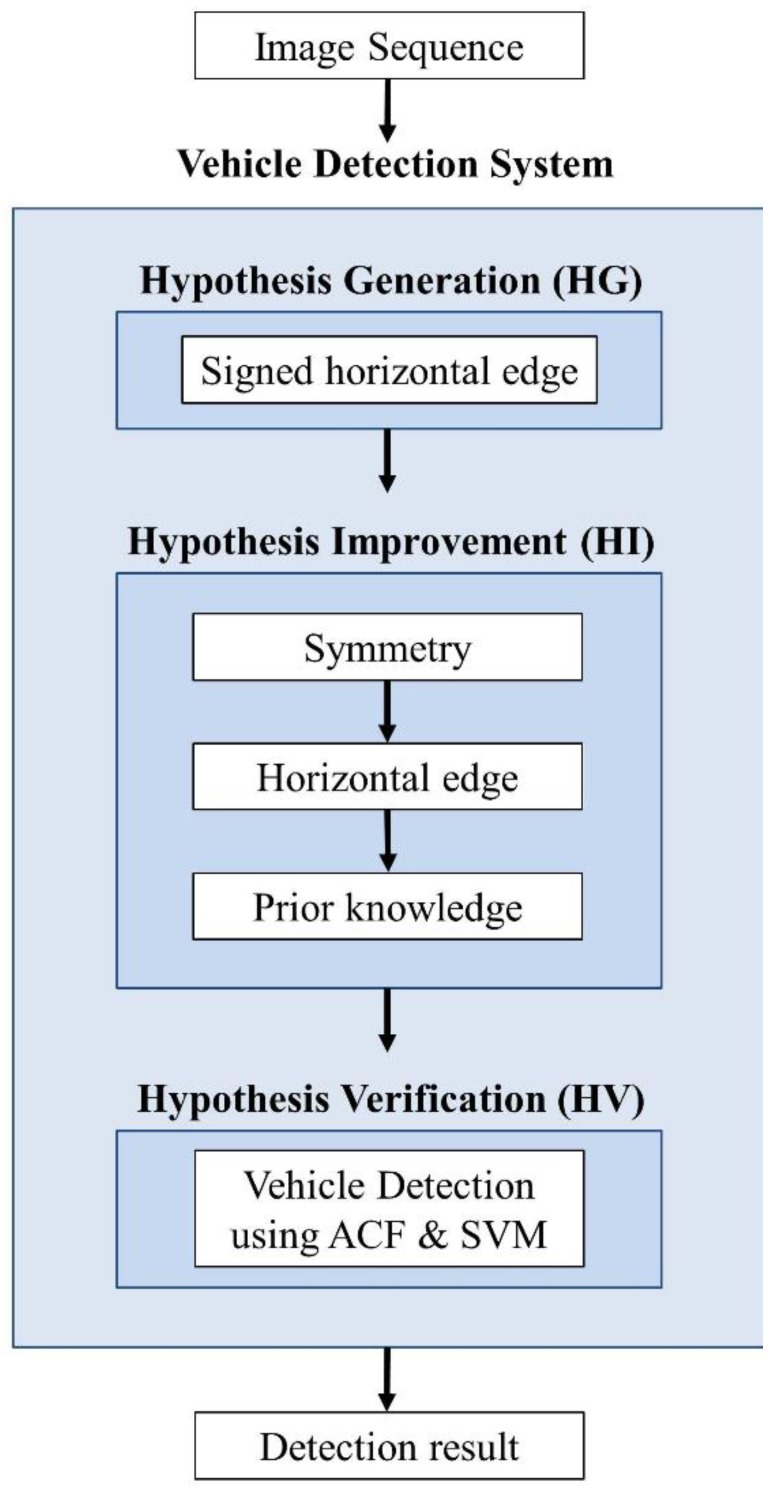
Flow chart of the proposed vehicle detection system.

The proposed system is outlined in Figure 3; as shown in the figure, the HI is positioned between the HG and the HV. In the HG, a simple method named a signed horizontal edge is developed to provide good hypothesized windows. In the HI, the aspect ratios of the hypothesized windows are estimated by combining the symmetry and horizontal edges of the vehicles. In the HV, ACF is employed with SVM to test the hypothesized windows with estimated aspect ratios.

## 3. Proposed Method

**Figure 4:**
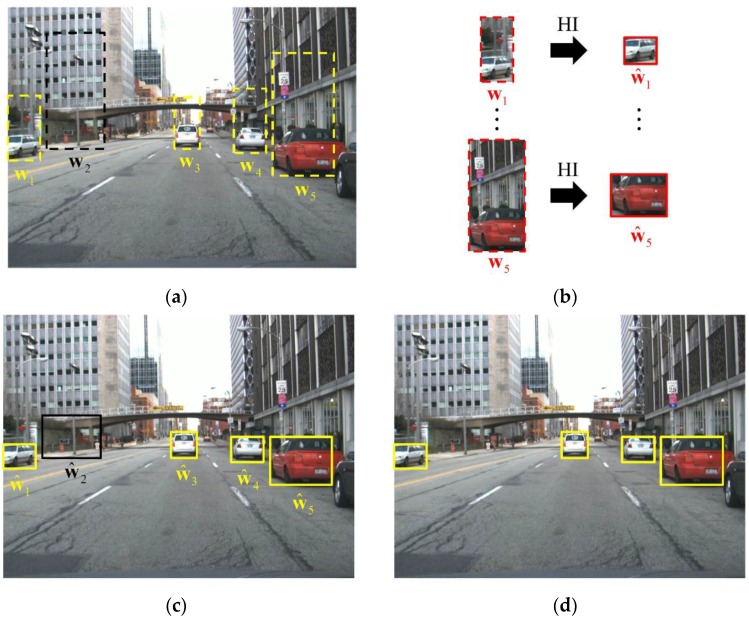
The framework of the proposed method: (**a**) shows the result of the HG by signed horizontal edges $\left(h_{i}=2w_{i}\right)$; (**b**) is the result of the estimation of the vehicle height; (**c**) shows the hypothesized windows given as the result of the HI; and (**d**) shows the vehicle detection results in the HV.

In this section, the three steps of the proposed method are explained. The results of the three steps are summarized, as in Figure 4. As in [28], the hypotheses for the vehicles are generated in the HG. Here, let us denote the i-th hypothesis for the vehicle as $w_{i}=\left(x_{i},y_{i},w_{i},h_{i}\right)$, where $\left(x_{i},y_{i}\right)$ denotes the left-lower position of the i-th hypothesis and $w_{i}$ and $h_{i}$ are the associated width and the height of the window, respectively. In the HI, the aspect ratio, or the equivalent height, of the hypothesized window $w_{i}$ is estimated. Initially, the height of the hypothesis is set to $h_{i}=2w_{i}$, which is long enough to include all kinds of vehicles, as shown in Figure 1. As in Figure 4a, the candidates of vehicle width are generated. Figure 4b shows the results of the estimation of vehicle height $h_{i}$ for the given vehicle width $w_{i}$. Let us denote the estimated value for the vehicle height by ${\hat{h}}_{i}$. Then, the i-th hypothesis is computed by ${\hat{w}}_{i}=\left(x_{i},y_{i},w_{i},{\hat{h}}_{i}\right)$. Figure 4c shows the hypothesized windows given as the result of the HI. Finally, ACF and SVM are used to test all of the hypothesized windows given from the HI. The vehicle detection results of the proposed method are shown in Figure 4d. 

### 3.1. Hypothesis Generation (HG)—A Signed Horizontal Edge Map

**Figure 5:**
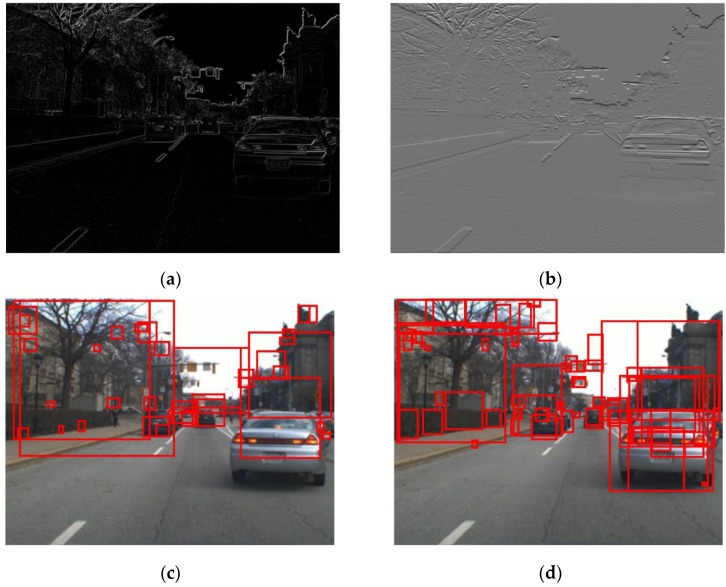
Hypothesis generation methods for VD: (**a**) Absolute edge image by [28]; (**b**) Signed horizontal edge image; (**c**) HG by absolute edge image [28]; and (**d**) HG by signed horizontal edge image.

An efficient hypothesis generation method for VD was reported by Alonso *et al.* in [28]. The method uses an absolute edge map defined by

(1) $$E\left(x,y\right)=\left|E_{h}\left(x,y\right)-E_{v}\left(x,y\right)\right|$$

where $E_{h}\left(x,y\right)$ and $E_{v}\left(x,y\right)$ represent the horizontal and vertical gradient images, respectively. Figure 5a,c shows an original vehicle image I(x,y) and the associated absolute edge map E(x,y) and the generated hypotheses. The absolute edge map method in [28] is very efficient but it has the drawback that the absolute edge map E(x,y) sometimes misses some weak horizontal edges such as vehicle shadows, degrading the VD performance. To avoid missing some weak horizontal edges such as shadow edges, the signed horizontal edge map computed by

(2) $$E_{s}\left(x,y\right)=I\left(x,y\right)*H,\;H=\left[\begin{array}{ccc} -1 & -2 & -1\\ 0 & 0 & 0\\ 1 & 2 & 1 \end{array}\right]$$

It is used as in Figure 5b. The signed horizontal edge map $E_{s}\left(x,y\right)$ takes into account both sign and magnitude of the horizontal edges and outperforms the absolute edge map E(x,y) in detecting the edges between shadows and roads since the shadows tend to be darker than the roads. Figure 5b,d show the same image I(x,y), and the associated edge $E_{s}\left(x,y\right)$ and the generated hypotheses. The signed horizontal edge map $E_{s}\left(x,y\right)$ outperforms the absolute edges image E(x,y) in the HG.

### 3.2. Hypothesis Improvement (HI)–Aspect Ratio Estimation

In this subsection, the symmetry of the vehicle images, the horizontal edges and the prior knowledge about the aspect ratio of the vehicles are combined to estimate the aspect ratio of the hypothesized windows provided by the HG. 

#### 3.2.1. Symmetry

**Figure 6:**
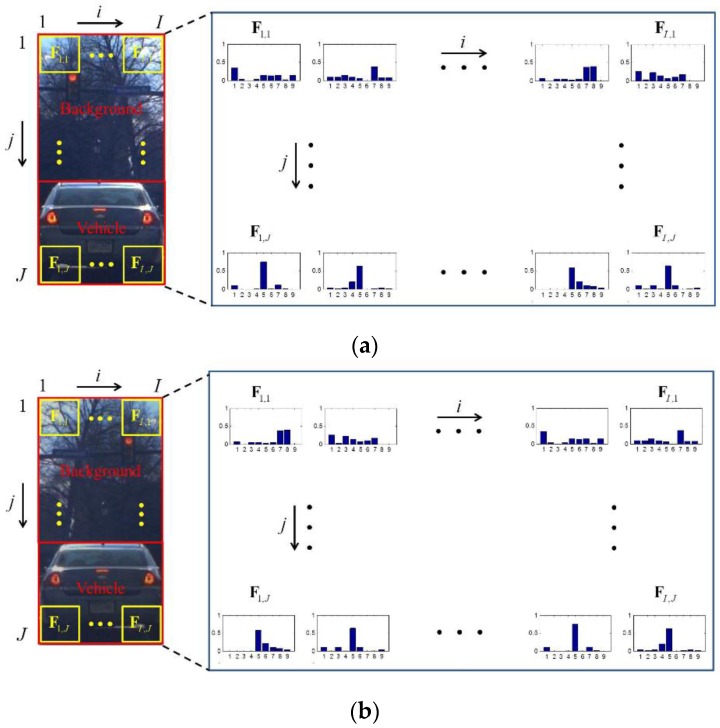
The procedure of using the symmetry: (**a**) is the HOG feature of the hypothesized window; and (**b**) is the HOG feature of the flipped hypothesized window (T=9).

The basic idea of this subsection is to exploit the fact that vehicles are symmetric while the backgrounds are not, as shown in Figure 6. The symmetry for each value in the y axis is computed as follows: 

(1) The given hypothesized window is flipped horizontally as in Figure 6, making a mirror image. Figure 6a,b show examples of the original image and the corresponding flipped image, respectively. For js that belong to vehicles, the two images are almost the same while, for js that belong to backgrounds, the two images are different from each other. 

(2) In order to quantify the symmetry of the given hypothesized window, the similarity between the hypothesized window and the mirror image is computed. Instead of intensity values, gradient values in images are used because they are more robust than intensity values under various illuminations. Thus, the HOG feature vector, is used. The HOG feature is a part of ACF and includes the gradient magnitude and orientation. The HOG feature vector for a hypothesized window can be denoted by $H=\left[F_{1,1},\;\cdots \;,\;F_{I,1},\;\cdots \;,\;F_{1,J},\;\cdots \;,\;F_{I,J}\right]\in {ℜ}^{TIJ}$; $F_{i,j}=\left[B_{i,j}^{1},\;\cdots \;,\;B_{i,j}^{T}\right]\in {ℜ}^{T}$ denotes the histogram of the (i,j) block, and $B_{i,j}^{t}$ denotes the sum of the gradient magnitudes according to the orientation bin t in the (i,j) block, where I and J are the numbers of column and row blocks of the window, respectively, as shown in Figure 6; T denotes the number of orientation bins in the HOG; i,j and t are the indices for I, J and T, respectively. 

(3) For the HOG of the hypothesized window $H=\left[F_{1,1},\;\cdots \;,\;F_{I,1},\;\cdots \;,\;F_{1,J},\;\cdots \;,\;F_{I,J}\right]\in {ℜ}^{TIJ}$ and the HOG of the associated flipped image $H^{F}=\left[F_{1,1}^{F},\;\cdots \;,\;F_{I,1}^{F},\;\cdots \;,\;F_{1,J}^{F},\;\cdots \;,\;F_{I,J}^{F}\right]\in {ℜ}^{TIJ}$, the similarity between the two HOGs is defined by

(3) $$\begin{array}{l} S=H\circ H_{F}=\left[\begin{array}{ccccccc} \underset{\in {ℜ}^{T}}{\underset{︸}{F_{1,1}\circ F_{1,1}^{F}}} & \cdots & \underset{\in {ℜ}^{T}}{\underset{︸}{F_{I,1}\circ F_{I,1}^{F}}} & \cdots & \underset{\in {ℜ}^{T}}{\underset{︸}{F_{1,J}\circ F_{1,J}^{F}}} & \cdots & \underset{\in {ℜ}^{T}}{\underset{︸}{F_{I,J}\circ F_{I,J}^{F}}} \end{array}\right]\\ =[\begin{array}{ccc} \underset{\in {ℜ}^{T}}{\underset{︸}{\left[\begin{array}{ccc} B_{1,1}^{1}\cdot B_{1,1}^{1F} & \cdots & B_{1,1}^{T}\cdot B_{1,1}^{TF} \end{array}\;\right]}} & \cdots & \underset{\in {ℜ}^{T}}{\underset{︸}{\left[\begin{array}{ccc} B_{I,1}^{1}\cdot B_{I,1}^{1F} & \cdots & B_{I,1}^{T}\cdot B_{I,1}^{TF} \end{array}\;\right]}} \end{array}\\ \;\begin{array}{cccc} \cdots & \underset{\in {ℜ}^{T}}{\underset{︸}{\left[\begin{array}{ccc} B_{1,J}^{1}\cdot B_{1,J}^{1F} & \cdots & B_{1,J}^{T}\cdot B_{1,J}^{TF} \end{array}\;\right]}} & \cdots & \underset{\in {ℜ}^{T}}{\underset{︸}{\left[\begin{array}{ccc} B_{I,J}^{1}\cdot B_{I,J}^{1F} & \cdots & B_{I,J}^{T}\cdot B_{I,J}^{TF} \end{array}\;\right]}} \end{array}]\\ =\left[\begin{array}{ccccccc} \underset{\in {ℜ}^{T}}{\underset{︸}{s_{1,1}}} & \cdots & \underset{\in {ℜ}^{T}}{\underset{︸}{s_{I,1}}} & \cdots & \underset{\in {ℜ}^{T}}{\underset{︸}{s_{1,J}}} & \cdots & \underset{\in {ℜ}^{T}}{\underset{︸}{s_{I,J}}} \end{array}\right]\in {ℜ}^{TIJ} \end{array}$$

where ∘ denotes component-wise multiplication; $s_{i,j}=\left[\begin{array}{ccc} B_{i,j}^{1}\cdot B_{i,j}^{1F} & \cdots & B_{i,j}^{T}\cdot B_{i,j}^{TF} \end{array}\right]\in {ℜ}^{T}$. The symmetry for the j-th row vector block can be quantified by

(4) $$m=\left[m_{1},\;\cdots \;,\;m_{J}\right],\;m_{j}=\sum_{i=1}^{I}{\left\|s_{i,j}\right\|}_{1}$$

**Figure 7:**
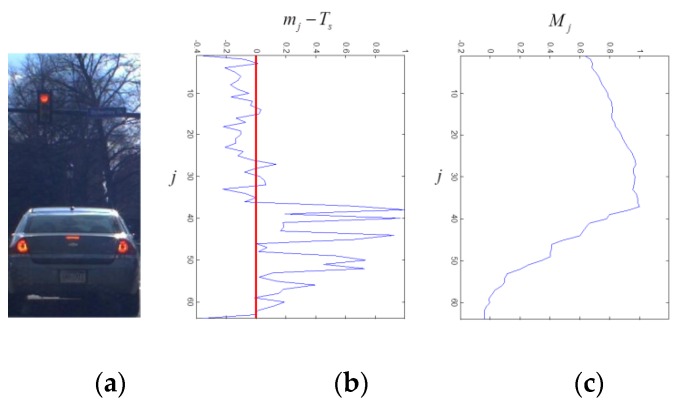
The result of estimating symmetry: (**a**) is the hypothesized window; (**b**) is the symmetry in terms of j; and (**c**) is the accumulated symmetry from bottom to top (I=8, J=64).

Finally, the symmetry is summed over all js (over all row blocks) and the accumulated symmetry is defined as

(5) $$M=\left[M_{1},\;\cdots \;,\;M_{J}\right],\;M_{j}=\sum_{t=0}^{J-j}\left(m_{J-t}-T_{s}\right)$$

where $T_{s}$ is a median value of the symmetry vector m. That is, the accumulated symmetry $M_{j}$ for the j-th vector row block is the sum of the symmetry values of m from the bottom to the j-th vector row block. Figure 7 shows the computation results of the accumulated symmetry for vehicle images. In Figure 7b, the symmetry is depicted for different js. Since the vehicle region has high symmetry, the background region has low symmetry, and $T_{s}$ is a median value of the symmetry vector m, the j-th row vector block corresponding to the vehicle height has a maximum accumulated symmetry as shown in Figure 7c. 

#### 3.2.2. Horizontal Edge

**Figure 8:**
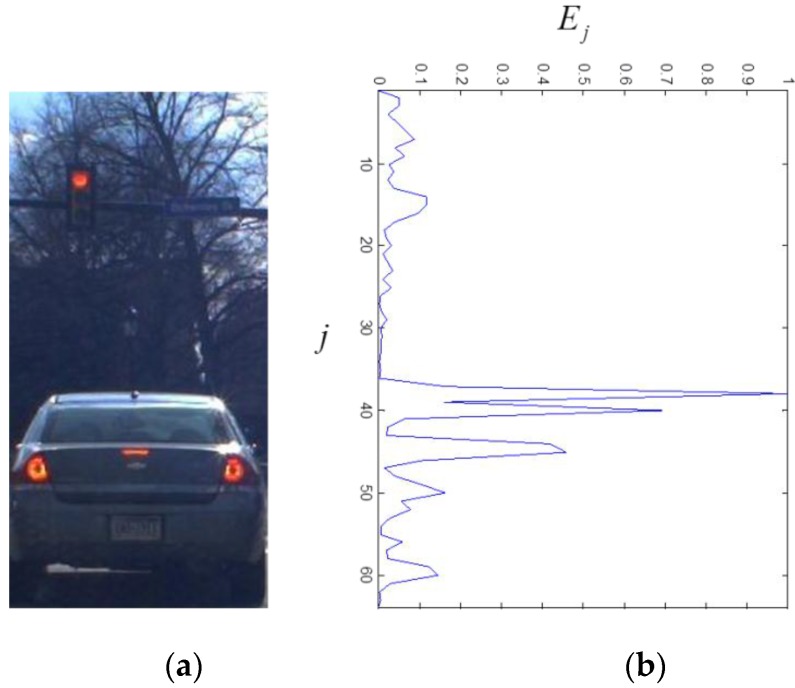
The result of the horizontal edge: (**a**) is the hypothesized window; and (**b**) is the horizontal edge in terms of j.

In addition to the symmetry of the vehicles, the horizontal edge is also an important cue that we can use to estimate the vehicle height. The horizontal edge is also computed using the HOG feature vector H. Figure 8 shows the result of the horizontal edge detection. The amount of the horizontal edge is defined by

(6) $$E=\left[E_{1},\;\cdots \;,\;E_{J}\right],\;E_{j}=\sum_{i=1}^{I}B_{i,j}^{t_{0}}$$

where $t_{0}$ denotes the bin for the horizontal orientation. For simplicity, E is also called the horizontal edge. In Figure 8b, the j corresponding to the vehicle height has the highest magnitude of the horizontal edge due to the intensity difference between the vehicle and the background.

#### 3.2.3. Prior Knowledge about Aspect Ratio

**Figure 9:**
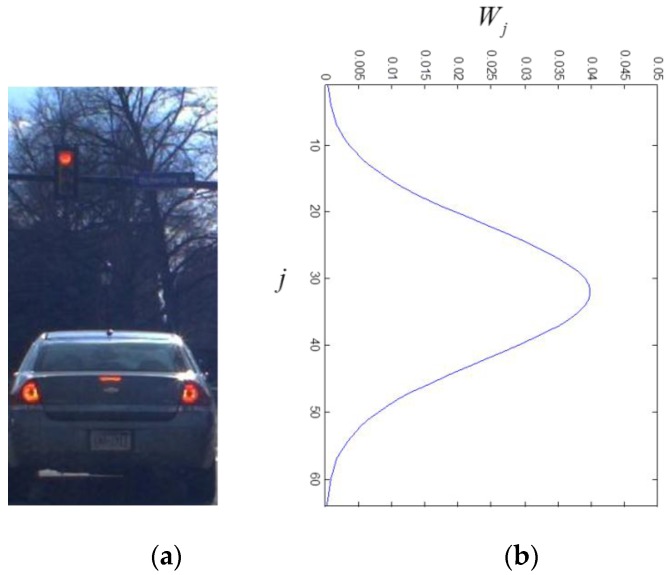
The prior knowledge match degree: (**a**) is the hypothesized window; and (**b**) is the prior knowledge match degree in terms of j (σ=10).

Finally, our prior knowledge about aspect ratio is used to fine-tune the heights of vehicles. As shown in Figure 1, it is unusual for the vehicle aspect ratio to be less than 0.5 or larger than 1.5. Thus, we model the vehicle aspect ratios using a Gaussian distribution, as shown in Figure 9. The degree to which the estimated aspect ratio matches our prior knowledge is used to reduce false estimations of the vehicle aspect ratio. Here, prior knowledge match degree is defined by

(7) $$\begin{array}{l} W=\left[W_{1},\;\cdots \;,\;W_{J}\right]\\ W_{j}=N\left(j|\frac{J}{2},\sigma \right)=\frac{1}{\sigma \sqrt{2\text{π}}}\exp\left(-\frac{{\left(j-J/2\right)}^{2}}{2\sigma^{2}}\right) \end{array}$$

where N(·) denotes a Gaussian distribution. The mean of the vehicle aspect ratio is set to one. Figure 9 shows the distribution of the prior knowledge match degree.

#### 3.2.4. Estimating Vehicle Height

Three measures, the accumulated symmetry $M_{j}$, the horizontal edge $E_{j}$, and the prior knowledge match degree $W_{j}$, are combined to define a score for the vehicle height as

(8) $$T=\left[T_{1},\;\cdots \;,\;T_{J}\right],\;T_{j}=M_{j}\cdot E_{j}\cdot W_{j}.$$

**Figure 10:**
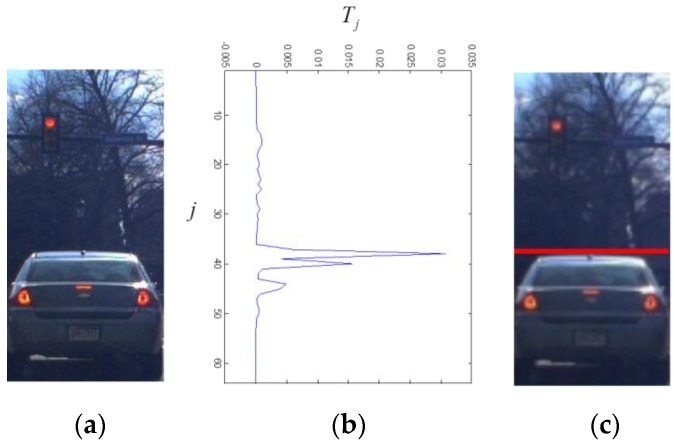
The results of estimating vehicle height: (**a**) is the hypothesized window; (**b**) is the total score for vehicle height estimation; and (**c**) shows the estimated vehicle height represented by the red line.

Figure 10 shows the final height score $T_{j}$ of a window for different values of j. Using the height score, the vehicle height is estimated as in Equation (9) below:

(9) $$\hat{h}=h\cdot \frac{J-\hat{j}}{J},\;\hat{j}\;=\arg\underset{j}{\max}T_{j}.$$

In Figure 10c, the estimated height of a given vehicle is marked by a red line. As shown in the figure, the estimated height is very close to the ground truth.

### 3.3. Hypothesis Verification (HV)

**Figure 11:**
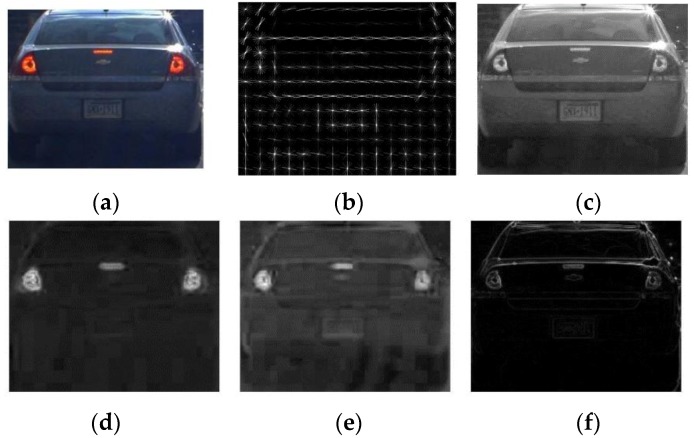
ACF channels for the image (**a**): (**b**) is HOG; (**c**) is L color space in LUV; (**d**) is U color space in LUV; (**e**) is V color space in LUV; (**f**) is normalized edge.

In the HV, ACF and SVM are applied to the fine-tuned windows obtained from the HI and the vehicle verification is conducted. ACF uses the five channels: normalized edge, HOG and LUV color channels. Figure 11 shows the ACF channels for the vehicle image. The channels are divided into 4 × 4 blocks and pixels in each block are summed [16]. The features extracted from ACF are trained by a linear SVM [29]. Finally, the trained SVM is used to detect the vehicles for the fine-tuned windows obtained from the HI.

## 4. Experiment

In this section, experiments are conducted to compare the performance the proposed method with that of the previous two methods. In Table 1, the HG, HI and HV of the competing algorithms are summarized. The first two algorithms denoted by “SW” and “Alonso” are the existing ones. Here, “SW” means a sliding window approach in [30] and “Alonso” means the algorithm in [28]. In “SW”, the aspect ratio is set to 1. The last one denoted by “SHE + VH” is the proposed method. Here, “SHE” means the signed horizontal edges and “VH” means the proposed aspect ratio estimation. 

**Table 1:** The mean absolute errors with the proposed method and previous methods.

Methods	HG	HI	HV
Previous method 1 (SW)	Sliding window	×	ACF + SVM
Previous method 2 (Alonso)	Absolute edge image	Peaks of edges	ACF + SVM
Proposed method (SHE + VH)	Signed horizontal edge image	Symmetry, Horizontal edge	ACF + SVM

The proposed methods are evaluated on three aspects: (1) the aspect ratio estimation; (2) the vehicle detection; and (3) the computation time. First, the aspect ratio estimation is considered. A total of 11,021 vehicles from the Pittsburgh dataset are used to evaluate the performance of the aspect ratio estimation. In Table 2, the previous methods and the proposed methods are compared in terms of the mean absolute error (MAE) between the true and estimated aspect ratios, which is defined by

(10) $$\frac{1}{N_{G}}\sum_{i=1}^{N_{G}}\left|R_{E}^{i}-R_{G}^{i}\right|$$

where $N_{G}$ is the number of vehicles; $R_{G}^{i}$ and $R_{E}^{i}$ are the true and estimated aspect ratios for the i-th sample, respectively. In Table 2, the MAE is evaluated for different types of vehicles: sedans, Sport Utility Vehicles (SUVs), trucks and busses. For sedans, trucks, and buses, the proposed method has a lower MAE than “SW” and “Alonso”. For SUVs, however, the proposed method underperforms compared to the previous methods. The reason for that is that the aspect ratio of a SUV is close to one and the fixed aspect ratio of one is better than the aspect ratio estimation. Overall, the proposed method demonstrates the lowest MAE among the competing methods and it means that the proposed method generates more accurate hypothesized windows than the previous methods do.

**Table 2:** The mean absolute errors (MAE) with the proposed and previous methods.

Vehicle	Number of Vehicles	Previous Method1 (“SW”)	Previous Method2 (“Alonso”)	Proposed Method (“SHE + VH”)
Sedan	5982	0.1425	0.1044	0.1014
SUV	4350	0.0694	0.0635	0.0902
Truck	390	0.3087	0.1844	0.0961
Bus	299	0.1415	0.3438	0.1309
Total	11,021	0.1656	0.1740	0.1047

**Figure 12:**
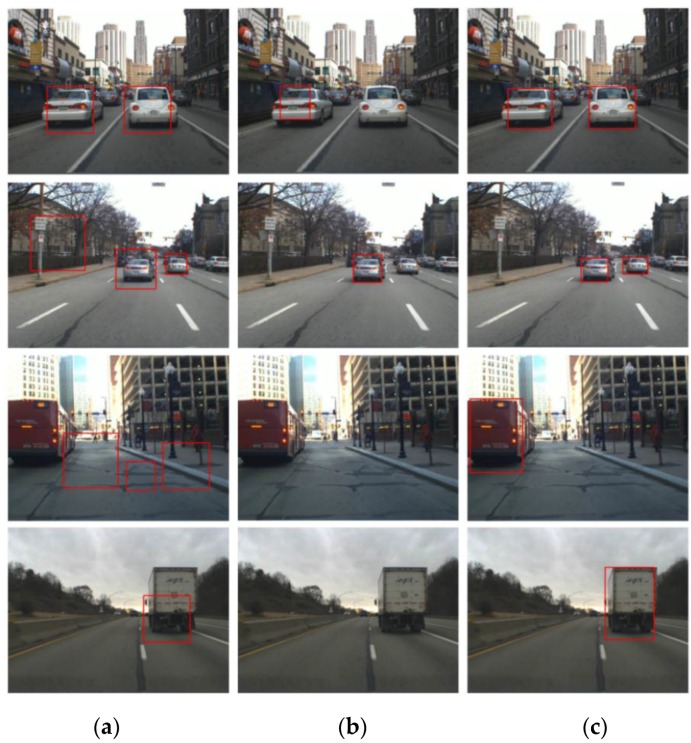
The vehicle detection result of (**a**) “SW”; (**b**) “Alonso”; and (**c**) “SHE+VH”.

Second, the proposed method is evaluated in terms of the VD performance. In Figure 12, the VD results of the previous methods and proposed methods are compared. From the figure, it can be seen that the bounding boxes of the proposed methods fit the vehicles more accurately than those of the previous methods. Further, the previous methods produce some false positives and miss some vehicles, while the proposed method detects the vehicles successfully. To qualitatively evaluate the detection performance, two measures are introduced: the PASCAL measure [31] and the average overlapping ratio (AOR).

The PASCAL measure considers a detection to be correct if the PASCAL measure r between the detection result $B_{D}$ and ground truth $B_{T}$ defined by

(11) $$r=\frac{\mathrm{area}\left(B_{D}\cap B_{T}\right)}{\mathrm{area}\left(B_{D}\cup B_{T}\right)}$$

exceeds a threshold $T_{r}$, where $B_{D}\cap B_{T}$ denotes the intersection between the detection result and ground truth, and $B_{D}\cup B_{T}$ denotes their union. In this experiment, the threshold $T_{r}$ is set to 0.55. Using the PASCAL measure, the true positive rate (TPR), the false positive per image (FPPI), and, subsequently, the Receiver operating characteristic (ROC) curve are evaluated. In addition to them, another measure, AOR, is proposed in this paper. The AOR is defined by

(12) $$\mathrm{AOR}=\frac{\sum_{i=1}^{N_{D}}r_{i}I\left[r_{i}>T_{r}\right]}{\sum_{i=1}^{N_{D}}I\left[r_{i}>T_{r}\right]}$$

**Figure 13:**
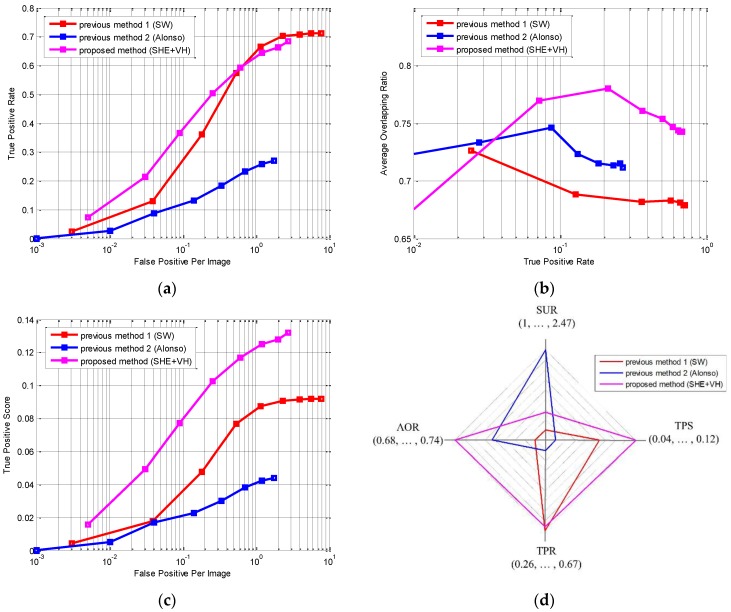
The detection performance in terms of (**a**) FPPI (false positive per image) *vs*. TPR (true positive rate); (**b**) TPR *vs.* AOR (average overlapping ratio); (**c**) FPPI *vs.* TPS (true positive score); and (**d**) SUR (speed-up ratio) *vs*. TPS *vs.* TPR *vs*. AOR when FPPI is 1.

It represents the accuracy of true positive detection, where $N_{D}$ is the number of the detected vehicles; $r_{i}$ is the PASCAL measure for the i-th vehicle detection; and I(⋅) is an indicator function that returns to one if the argument is true and zero otherwise. Using the TPR and AOR, we can define the true positive score (TPS) by

(13) $$\mathrm{TPS}=\mathrm{TPR}\cdot \left(\mathrm{AOR}-T_{r}\right)=\left(\frac{1}{N_{G}}\sum_{i=1}^{N_{D}}I\left[r_{i}>T_{r}\right]\right)\cdot \left(\frac{\sum_{i=1}^{N_{D}}r_{i}I\left[r_{i}>T_{r}\right]}{\sum_{i=1}^{N_{D}}I\left[r_{i}>T_{r}\right]}-T_{r}\right)=\frac{1}{N_{G}}\sum_{i=1}^{N_{D}}\left(r_{i}-T_{r}\right)I\left[r_{i}>T_{r}\right]$$

where $N_{G}$ is the number of vehicles. TPS reflects both TPR and AOR and it represents the true detection rate and accuracy simultaneously. In Figure 13, the detection performances of the proposed and previous methods are compared in terms of the TPR, FPPI, ROC and AOR. In the experiment, the size of the images is 320 × 240 and only the vehicles covering more than 30 pixels are considered as true targets. Figure 13a is the ROC. From the figure, the proposed method demonstrates better detection performance than the previous methods. In Figure 13b, the AOR (detection accuracy) is depicted against TPR (detection rate). This figure clearly shows that the proposed method outperforms the previous two methods in detection accuracy (AOR) when the detection rates (TPR) are the same. In Figure 13c, the detection performance is compared in terms of the TPS and FPPI. The TPS is the combination of the detection accuracy and rate. The proposed method demonstrates much higher TPS than the previous methods, meaning that the proposed method detects the vehicles better and more accurately than the previous methods simultaneously. In Figure 13d and Table 3, three competing methods are compared in terms of the speed-up ratio (SUR) [32], TPS, TPR and AOR when the FPPI is set to 1. SUR means how much faster the algorithm runs in comparison with the exhaustive search “SW” and it is defined as

(14) $$\mathrm{SUR}\text{=}\frac{processing\;time\;of\;“SW”}{processing\;time\;of\;an\;algorithm}$$

**Table 3:** The overall performance of the proposed and previous methods (when FPPI is 1).

Methods	TPS	TPR	AOR	SUR
Previous method 1 (SW)	0.0872	**0.665**	0.6812	1
Previous method 2 (Alonso)	0.0425	0.2574	0.715	**2.4736**
Proposed method (SHE + VH)	**0.1249**	0.6436	**0.744**	1.3243

From the figure and table, the proposed method runs 1.87 times slower than “Alonso” but it achieves much better performance than “Alonso” in the other three measures. Compared with “SW”, the proposed method runs 1.32 times faster and it achieves much better performance in TPS and AOR. Its TPR is almost the same as that of “SW”. Thus, it is evident that the proposed method is attractive both in detection rate and accuracy even though it is computationally slightly more expensive than “Alonso”.

## 5. Conclusions

In this paper, a precise new on-road vehicle detection system has been proposed. In situations that require the vehicle position and size, accurate vehicle detection is very important. For accurate vehicle detection, the signed horizontal edge map was proposed in the HG and the aspect ratio of the vehicle windows was estimated in the HI. The windows from the HI were provided to the HV composed of the ACF and SVM, and good VD performance was obtained. 

Finally, a new measurement was proposed to test the accuracy of the proposed vehicle detection method. In the experiment, the proposed method was compared with the previous methods in terms of the TPR, FPPI, ROC, AOR, and SUR. The validity of the proposed method was proven through experimentation. 
